# Open vs. Closed Skill Sports and the Modulation of Inhibitory Control

**DOI:** 10.1371/journal.pone.0055773

**Published:** 2013-02-13

**Authors:** Chun-Hao Wang, Che-Chien Chang, Yen-Ming Liang, Chun-Ming Shih, Wen-Sheng Chiu, Philip Tseng, Daisy L. Hung, Ovid J. L. Tzeng, Neil G. Muggleton, Chi-Hung Juan

**Affiliations:** 1 Institute of Neuroscience, National Yang-Ming University, Taipei, Taiwan; 2 Institute of Cognitive Neuroscience, National Central University, Jhongli, Taiwan; 3 Office of Physical Education, National Central University, Jhongli, Taiwan; 4 Graduate School of Human Sexuality, Shu-Te University, Kaoshiung, Taiwan; 5 Laboratories for Cognitive Neuroscience, National Yang-Ming University, Taipei, Taiwan; 6 Institute of Linguistics, Academia Sinica, Taipei, Taiwan; 7 Institute of Cognitive Neuroscience, University College of London, London, United Kingdom; University of Milan, Italy

## Abstract

**Background:**

Inhibitory control, or the ability to suppress planned but inappropriate prepotent actions in the current environment, plays an important role in the control of human performance. Evidence from empirical studies utilizing a sport-specific design has shown that athletes have superior inhibitory control. However, less is known about whether this superiority might (1) still be seen in a general cognitive task without a sport-related context; (2) be modulated differentially by different sporting expertise (e.g., tennis versus swimming).

**Methodology/Principal Findings:**

Here we compared inhibitory control across tennis players, swimmers and sedentary non-athletic controls using a stop-signal task without a sport-specific design. Our primary finding showed that tennis players had shorter stop-signal reaction times (SSRTs) when compared to swimmers and sedentary controls, whereas no difference was found between swimmers and sedentary controls. Importantly, this effect was further confirmed after considering potential confounding factors (e.g., BMI, training experience, estimated levels of physical activity and VO2max), indicative of better ability to inhibit unrequired responses in tennis players.

**Conclusions/Significance:**

This suggests that fundamental inhibitory control in athletes can benefit from open skill training. Sport with both physical and cognitive demands may provide a potential clinical intervention for those who have difficulties in inhibitory control.

## Introduction

The ability to suppress ongoing or planned but inappropriate actions in a given situation requires inhibitory control [1], [2]. Hence, inhibitory control plays an important role in the selection of appropriate behaviors in daily life. More specifically, inhibitory control is also associated with successful sporting performance [3]. Thus, it may be that inhibitory control is better developed in athletes, suggesting a higher degree of behavioral performance, relative to those who are non-athletic. However, few studies have investigated the effect of sports experience on general cognitive traits (e.g., inhibitory control) [3], [4], and in particular the comparison between various sport categories [4]. Following in this vein, the present study explores the differences in inhibitory control between athletes and non-athletes as well as between athletes from two different sport categories (i.e., tennis and swimming).

Studies have demonstrated that frontal-dominated cognitive functions, such as executive control, conflict solving, and inhibitory control, can benefit from both enhanced aerobic fitness [5]–[7] and extensive sport training [3], [7]. In terms of inhibitory control in athletes, prior studies which have shown that athletes such as baseball players [8]–[10]; basketball players [10]; and fencers [7], [11], responded faster or committed fewer errors when compared to non-athletes in Go/No-go tasks, where subjects are required to refrain from responding to the no-go signals. For example, Di Russo et al. [11] found that fencers responded faster with respect to non-athletes selectively in the condition with response inhibition rather than in the simple response condition, suggesting that sporting training may result in enhanced response inhibition. In agreement with this, evidence from studies investigating eye movements, in tasks such as the anti-saccadic task involving response inhibition [12], showed that table tennis players [13], [14], basketball players [15] and elite shooters [16] had shorter anti-saccadic latencies or less anti-saccadic errors compared to non-athletes. This evidence from manual and ocular responses suggests that inhibitory control might be enhanced via extensive practice of at least in some types of sport training.

Although previous research has shown that athletes display superiority in inhibitory control, less is known about whether this can be modulated differentially across sport categories [4] as a result of the differences in required cognitive and motor demands that differ from one sport category to another [17]–[19]. In general, sports may be categorized into two types: open skill and closed skill sports. Open skill sports are defined as those in which players are required to react in a dynamically changing, unpredictable and externally-paced environment (e.g., basketball, tennis, fencing and etc.) [20]. By contrast, closed skill sports are defined as those in which the sporting environment it is relatively highly consistent, predictable, and self-paced for players (e.g., running, swimming) [4], [20]. Athletes from open skill sports may develop more flexibility in visual attention, decision making and action execution [21], relative to athletes from closed skill sports. This rationale can be supported by meta-analysis studies that showed that athletes from open skill sports (also referred to as interceptive or strategic sports) performed better in cognitive tasks than those from closes skill sports (or static sports), indicating the importance of comparing different types of sport [4], [22].

Another factor necessary to consider may be any sport-related context in the cognitive task. For example, one study investigating response inhibition has reported that athletes from open skill sports (baseball players) performed better compared to those belonging to static sports (track-and-field or gymnastics), but this superiority in inhibitory control occurred only in sport-specific experimental designs [8]. Therefore, it is also of interest to examine whether any difference in inhibitory control across sport categories can also be found by means of a task without a sport-specific declarative or procedural knowledge [4].

While the Go/No-go tasks was used in previous investigations of athletic superiority in inhibitory control [8], [11], the present study employing a stop-signal task which has been used in a number of previous studies investigating inhibitory control [18], [23]–[28]. In this task, there are two types of signals: (1) the go signal, which a response has to be made as soon as possible; (2) and the stop signal, which, when presented, requires the response to be withheld. One critical manipulation is the stop-signal delay (SSD) which is the interval between the go signal and the stop signal: the longer the SSD, the more difficult it is to inhibit responding, resulting in higher error rates [23], [29]. This manipulation allows calculation of the stop-signal reaction time (SSRT), a measure of the time required to inhibit a prepotent responses. Studies using this task have observed longer SSRTs in violent offenders [30], children with attention deficit hyperactivity disorder (ADHD) [31], and patients with Parkinson’s disease [32] when compared to their normal counterparts, suggesting poorer inhibitory control in these kinds of subjects.

Here we used the stop-signal task without a sport-specific design to investigate the effect of different categories of sporting experiences on inhibitory control. A racket sport (tennis) was chosen as the sport for the open skill sport group due to its requirement for superior motor control, fast interceptive actions, hand-eye coordination and a high perception-action demand [19], [33]. This sport also requires that players inhibit action within a very limited period if the ball is going out of play [34]. Additionally, Tsai [35] demonstrated that racket sports have the capacity to improve inhibitory control performance in children with developmental coordination disorder (DCD). Swimmers were chosen as the closed skill sport group due to its stable, predictable training environment, and because the skills in swimmers are less affected by the environment [4], [20], which may consequently result in less enhancement in cognitive skills relative to open skill sports. Sedentary controls were recruited from those who reported having no historical specialty in any sports and did not partake in regular exercise at least in the 6 months prior to the study. Because better cognitive-motor performance might benefit from activities requiring both aerobic and cognitive demands [7], we predicted tennis players would commit fewer errors response to stop-signal or have shorter SSRT than sedentary controls, whereas this superiority might be less significant in swimmers.

## Methods

### Ethics Statement

A local ethical committee (Institutional Review Board of the Kaohsiung Medical University Chung-Ho Memorial Hospital, Kaohsiung, Taiwan) approved the experiment. All subjects provided written informed consent prior to participating.

### Participants

Sixty male students were recruited from a university in northern Taiwan. Table 1 summarizes the subjects’ characteristics. Of these, twenty students were members of the varsity tennis team (aged 20.23±2.39 years, with tennis experience of 3 to 11 years, mean 5.50±2.80 years; ongoing training program: 3 hours a day, 3 or more days a week; 6 singles players, 5 doubles players, and 9 who played both types were included), while another twenty students belonged to the varsity swim team (aged 19.31±0.75 years, with experience of 2.5 to 9 years, mean 4.85±1.64 years; ongoing training program: 2.5 hours a day, 5 days a week; with a T30 swimming test score range from 1550 m to 1980 m). We found no difference in sporting experience between the athletic groups in terms of years of training [*t*(39) = .89, *p* = .381, *d* = 0.28]. The remaining twenty students reported no historical specialty in any sport/exercise and were sedentary at the time of the study (aged 20.92±2.33 years). Additionally, all of the athletes who participated in the study were qualified for the second level of National Intercollegiate Athletic Games in Taiwan which is equivalent to one level below a professional standard (for example, Taiwan tennis professionals are ranked from 59 to 1923 in the ATP world tour rankings). Because body mass index (BMI) is reported to be negatively associated with inhibitory control [36], we also controlled for BMI across the groups. All subjects had normal or corrected-to-normal visual acuity and were right-handed. No individuals reported having a history of neurological problems or cardiovascular diseases, nor were any taking any medications that affect cognitive functions.

**Table 1:** Group means (±SD) of the characteristics of the tennis players, swimmers, and sedentary controls.

	Tennis players (n = 20)	Swimmers (n = 20)	Sedentary Controls (n = 20)
Age (year)	20.70±2.43	19.31±0.75	20.40±2.09
Height (m)	1.74±0.06	1.70±0.06	1.73±0.06
Weight (kg)	69.65±9.64	63.99±7.47	67.13±10.04
BMI (kg/m2)	22.75±2.13	22.09±2.75	22.24±3.31
Kilocalorie expenditure (Kcal/d)	2703.79±394.85***	2761.63±389.59***	2177.27±332.04
VO_2max_ (mL*kg^−1^*min^−1^)	55.60±2.40***	55.05±2.41***	43.85±2.32
Training experiences	5.50±2.80	4.85±1.64	–

Note: *** denotes significantly different from controls at *p*<.001; – denotes no training experience.

### Instruments

This subsection will describe the instruments used in the present study, including the 7-day physical activity recall questionnaire, aerobic fitness evaluation, apparatus, and stop-signal task.

### Estimation for Levels of Physical Activity

To evaluate and quantify the subjects’ level of physical activity, a 7-day physical activity recall questionnaire was adapted from Sallis et al. [37], which has been shown to objectively estimate and quantify the participants’ level of physical activity [38]. In this questionnaire, the experimenter instructs the participants to recall their physical activities in the past 7 days, which can help estimate the time (hours) spent at different levels (e.g., light, moderate, high, intense, and sleep) of physical activity. Each level of intensity was indicated by a metabolic equivalent (MET, 1MET = 1 kcal/kg/hour): sleep = 1MET, light = 1.5 METs [24 hours – (sleep+moderate+moderate+high+intense)], moderate = 4 METs (e.g., golf, flexibility), high = 6 METs (e.g., doubles tennis, dancing), intense = 10 METs (singles tennis, swimming, jogging). The Kcal expenditure is calculated by the formula: *Kcal/day = Total physical activity (METs)/7×weight (kg)*. Thus, this questionnaire could successfully screen unwanted subjects and categorize subject groups. Additionally, because this questionnaire allowed the application of relatively strict screening criteria, it helped to ensure that athletic groups did not differ in their level of physical activity from control groups that would potentially bias the results. Hence, athletes and sedentary controls were excluded according to the following two criteria: (1) for the athletic groups: those who engaged in training programs less than three times per week; (2) for the control group, those who spent more than one hour per week exercising at the intensity of moderate or higher.

### Aerobic Fitness Estimation

Because aerobic fitness is demonstrated to be positively associated with inhibitory control [5], the current study employed a non-exercise formula to estimate the subjects’ VO_2max_, an index of aerobic fitness [39]. This formula has the capacity to provide a good prediction of the actual VO_2max_ (*R* = .93) in adults aged from 18–65 years [39], and can be used to estimate the state of athletes’ fitness [7]. Descriptive characteristics for subjects’ (age, height, weight, gender and BMI) were also collected. In addition, the Physical Activity Rating (PA-R) (ranging from 0–10 points) [39] and the Perceived Functional Ability (PFA) (ranging from 2–26 points) [40] questionnaires were used to survey the self-reported ability to walk, jog, or run at 1 and 3 miles (PFA) [e.g., Question: How fast could you cover a distance of 3-miles and NOT become breathless or overly fatigued? Be realistic. Answer: Number 11. I could jog the entire distance at a fast pace (8 minutes per mile)], and physical activity level for the previous 6 months (PA-R) [e.g., Question: Select the number that best describes your overall level of physical activity for the previous 6 months. Answer: 5 = vigorous activity: run 1 mile to less than 5 miles per week or spend 30 minutes to less than 60 minutes per week in comparable physical activity as descried above]. After collecting the required information, the levels of aerobic fitness were quantified by the formula: *VO2_max_ (mlkg^−1^* *min^−1^) = 48.073+(6.178×Gender; female = 0, male = 1) – (0.246×Age) – (0.619×BMI)+(0.712×PFA)+(0.671×PA-R)* [7], [39]. This helps to avoid potential confounding factors which might bias the results.

### Apparatus

Testing took place in a sound attenuated room. Stimuli were presented on a 17-in CRT screen using a video resolution of 800×600 pixels and a vertical refresh rate of 100 Hz. Subjects were seated 50 cm in front of the screen which was positioned at eye level. The task was programmed in E-prime (Psychology Software Tools, Inc, PA, USA).

### Stop-signal task

This task was adapted from previous studies [18], [23], [24]. In the stop-signal task, the stop signal delay (SSD) can be manipulated by adjusting the time between the onset of the go stimulus and the stop signal. The noncancelled rate denotes the error rate when the stop signal is presented but subjects fail to inhibit their response. The outcome of the race between the go and the stop processes is reflected by the inhibition function, describing the probability of responding for a given a stop signal delay in accordance with the race model [2]. The stop signal reaction time (SSRT) denotes the latency of the stop process and it is the most important dependent variable in the task. The SSRT can be measured from the observed distribution of RTs in no-stop signal trials in combination with the inhibition function [1]. In the present study, SSRTs for each SSD were estimated using the integration method and one summary SSRT was calculated by averaging the three SSRTs acquired from the three SSDs in our experiments [1], [28].

**Figure 1:**
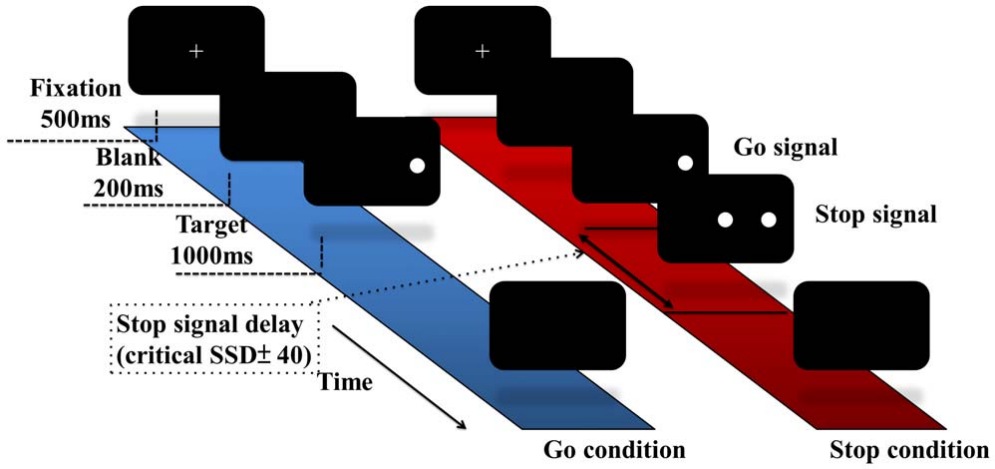
Stop-signal task procedure. The stop-signal task consisted of go and stop trials. All trials began central fixation. Following offset of the central fixation, a white peripheral dot was presented to the left or right of the fixation. On 25% of the trials (stop trials), the central fixation dot reappeared as an instruction to withhold responses.

Each trial of the stop-signal task presented here began with a 500 ms central fixation dot. Following the offset of this dot, a white target dot was presented to the left or right of the fixation at 9° eccentricity on the horizontal meridian (see Fig. 1). On 75% of the trials (go trials) subjects were required to make a key-press response with the index finger corresponding to the dot location, i.e. the left hand index finger when the dot was presented on the left and *vice versa*. On 25% of the trials (stop trials), the central fixation dot reappeared and served as a signal to withhold response to the peripheral target.

#### Stage 1: Obtaining the go RT

Every subject started with a session of the choice RT task (50 trials). They were asked to make the response correctly and as quickly as possible to a target which appeared either in the left or the right visual field with their corresponding index fingers. This was therefore essentially the stop signal task without any stop trials. The purpose of this session was to obtain each subject’s mean go RT and standard deviation without stop signals. The mean go RT plus two standard deviations was set as the maximum time limit for go RT trials in the subsequent sessions. If the subject did not respond quicker than this time restriction on a go trial, the trial was counted as a non-responding error and a warning beep would sound. This procedure has been demonstrated to effectively limit the strategy of intentionally slow responses that subjects sometimes use to avoid errors [18], [23], [24], [30].

#### Stage 2: SSD trials

A practice session consisting of 24 go trials and 8 stop trials was conducted following the Go RT section. The SSD was fixed at 170 ms in the stop trails in this section. The experimental trials were identical in structure apart from the SSD, as were the subsequent formal test session trials. After subjects performed the go time-limited session and the practice session, they in turn carried out a critical SSD session. The purpose of this session was to estimate each subject’s SSD at which their noncancelled rate would be around 50%. This session also helped to reduce the number of trials in the formal test sections. A tracking procedure was employed for acquiring the critical SSD. According to the previous studies [18], [24] and pilot experiments, the initial SSD was set at 170 ms. The SSD of each subject was adjusted until the accuracy on stop-trials reached 50%. The program monitored subject’s performance block by block, with each block including 32 trials. If the subject’s noncancelled rate was lower than 37.5%, the SSD was increased by 40 ms. If the noncancelled rate was higher than 62.5%, the SSD was decreased by 40 ms. A critical SSD could subsequently be computed that represented the time delay required for the subject to achieve a 50% success rate in inhibiting a response in the stop trials. Each subject’s critical SSD was determined when their noncancelled rate was within 37.5%–62.5% for two consecutive blocks and this typically took less than 500 trials.

#### Stage 3: Formal test section

After obtaining each subject’s mean go RT and critical SSD, they then carried out the formal stop-signal task. Three SSDs were presented to each subject based on their individual critical SSDs: (1) critical SSD, (2) critical SSD +40 ms, and (3) critical SSD –40 ms. For example, if a subject’s critical SSD was 130 ms (obtained in the critical SSD section), the other two SSDs were 90 ms and 170 ms. Three experimental blocks were presented for each condition, and each block included 48 trails, lasting approximately 4 minutes; the occurrence and order of the three stop signal presentation conditions were embedded randomly within each block.

### Procedure

**Figure 2:**
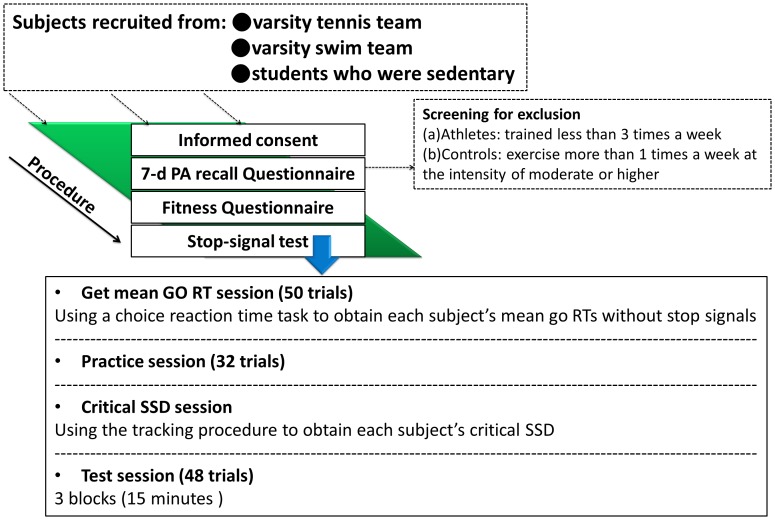
The procedures for the experimental sessions. Tennis players, swimmers and sedentary controls were firstly provided with informed consent, 7.-day physical activity recall questionnaire, and fitness questionnaire. Secondly, all eligible subjects took part in a stop-signal task consisted of three stages: get Go session, critical SSD session, and test session.

After providing informed consent, subjects took part in the experimental procedure that included two phases: the first phase consisted of the 7-day Physical Activity Recall and aerobic fitness questionnaires. The second phase comprised the stop signal task consisting of three stages. These were obtaining the mean GO RT, followed by SSD sessions, and finally the test session. The total duration of the experimental was approximately 40 minutes. The experimental procedure is illustrated in the Fig. 2.

### Data Analysis

#### Exclusion/inclusion

For the stop signal task performance, the following were filtered and were excluded from analysis: (1) non-response trials, (2) trials with responses to the wrong target, (3) trials with latencies more than 2 standard deviations above those obtained in the Go RT session.

**Figure 3:**
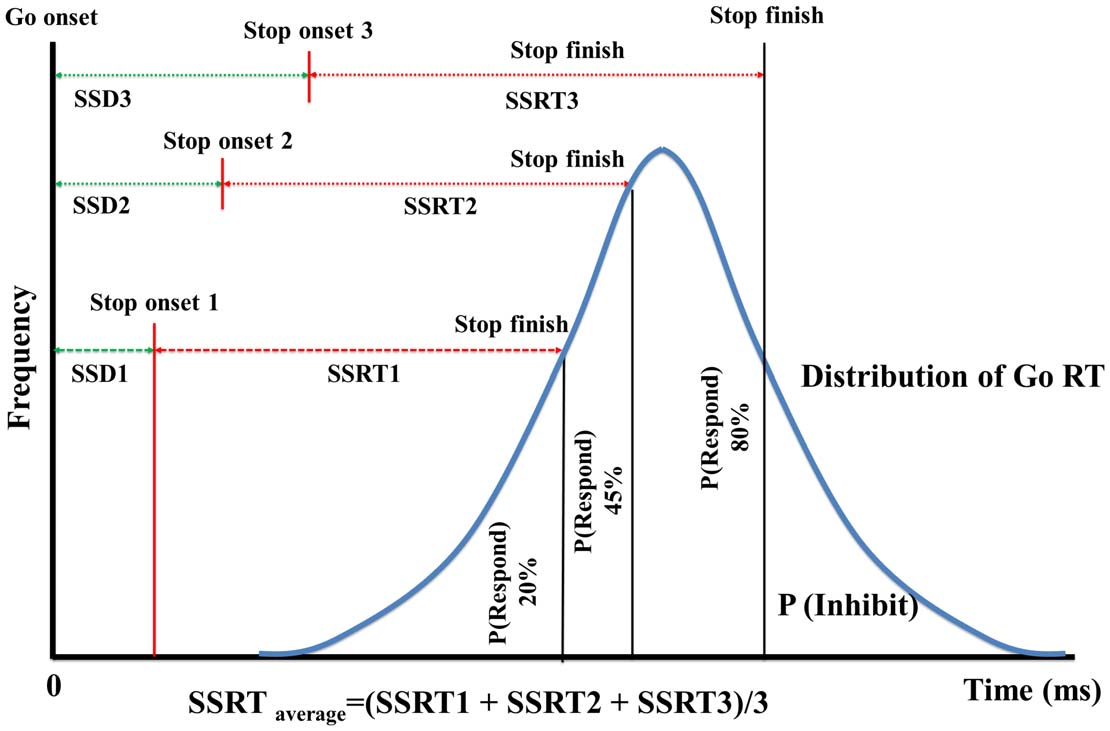
Stop-signal reaction time calculation. The figure illustrates the relation between stop signal delay, the stop signal reaction time and the distribution of go reaction times. The distribution of go reaction is integrated from the time of go signal presentation. For each stop signal delay, a probability of responding is obtained. If the stop signal delay of 50 ms resulted in an error rate = .20, this means that the end of the stop process should be at a point equal to 20% of the go RT distribution. If the point of 20% of the go RT distribution was 250 ms, so the observed SSRT would be 252–50 ms. The rest of the SSRT were calculated with the same procedure. A summary SSRT was acquired by averaging the observed three SSRTs that corresponded to 0.15<p(respond)<0.85 [28].

Stop signal reaction times (SSRT) were calculated using the distribution of go signal reaction times and the probability of responding for a given stop signal delay (the inhibition function) in accordance with the race model [2]. In the present study, SSRTs for each stop signal delay were estimated by using the integration method [5], [18], [24]. These were then averaged to obtain a summary SSRT (SSRT_average_ in Band et al. [28]’s terminology). We followed the method introduced by Logan [1], [2] to calculate the SSRT for each SSD. Briefly, if the noncancelled rate = *x*, at a given SSD, the stop processes must have finished at point *x* of the observed go RT distribution. The value of the *x* point minus SSD yields the SSRT. For example, if SSD = 130 ms, the noncancelled rate = 0.4, and the 40^th^ percentile RT of the observed go RT distribution = 330 ms, the observed SSRT will be 330–130 = 200 ms for this SSD. Finally, the SSRTs for each SSD were averaged for analysis. Fig. 3 illustrates an example how SSRT was acquired in an example subject.

#### Statistical analysis

All subjects’ characteristics were described using means and SDs, and group differences were submitted to a one-way ANOVA method with a post-hoc Bonferroni-corrected t test. A one-way ANOVA with a Bonferroni adjustment for multiple comparisons was conducted to analyze the filtered responses, go RTs without a stop signal, go RT trials with a stop signal, noncancelled RTs, SSRTs and error rates. The inhibition function data were analyzed using a two-way [3 (groups: tennis, swim, control)×3 (SSDs: critical SSD –40 ms, critical SSD, critical SSD+40 ms)] two-way mixed ANOVA with a Bonferroni adjustment for multiple comparisons. Moreover, because basic factors such as BMI, training experience, estimated levels of physical activity and VO2max may be related with SSRTs due to their associations with cognitive performance [4]–[7], [20], [36], we adopted a hierarchical stepwise regression approach which has the capacity to tease out confounding factors. This procedure evaluates each variable in turn on the basis of extent of correlation and builds the model by adding variables sequentially. The variable having highest correlation with the dependent variable would be added to the model first, then the second best or so on. Variables are added as long as *R^2^* is significantly increasing. In the present study, SSRT performance was used as the dependent variable, and BMI, training experiences, estimated levels of physical activity and VO2max, and groups were used as independent variables. In this model, the basic factors including BMI, training experience, estimated levels of physical activity and VO2max are entered in the first step. The group variables are then added in the second step. To represent the three-category variable “group”, we use two dummy variables. We let the “control” category be the reference category, and create two dummy variables: (1) TennisCon = 1 if a tennis player; 0 otherwise (2) SwimCon = 1 if a swimmer; 0 otherwise. Additionally, prior to testing this model, a bivariate Pearson correlation was performed to test the relationships between the variables (see Table 2). The level of significance was set at *p*≤.05. All analysis was completed with the SPSS 18.0 Software System.

**Table 2:** Correlation analysis for BMI, training experiences, estimated physical activity, estimated VO2max, group factors, and stop-signal reaction time.

Variables	1	2	3	4	5	6	7
1.BMI	–						
2. VO_2max_	−.23	–					
3.Training experiences	−.08	.72***	–				
4.Kilocalorie expenditure	.51***	.46**	.34**	–			
5.TenCon	.10	.49***	.47***	.25	–		
6.SwimCon	−.07	.43**	.32**	.34**	−.50***	–	
7.Stop-signal reaction time	.07	−.34**	−.27*	−.20	−.53***	.19	–

Note: ***denotes *p*<.001;

** denotes *p*<.01;

* denotes *p*<.05.

## Results

### Subject Demographics

Subject demographic data and their levels of physical activity are provided in Table 1. Demographic variables including age (*F*(2,57) = 2.36, *p* = .10, *η_p_^2^* = .08), height (*F*(2,57) = 2.40, *p* = .100, *η_p_^2^* = .08), weight (*F*(2,57) = 1.94, *p* = .153, *η_p_^2^* = .06) and BMI (*F*(2,57) = .31, *p* = .735, *η_p_^2^* = .01), did not differ between groups.

#### Estimation of levels of physical activity

There was a significant group difference for the estimated levels of physical activity (*F*(2,57) = 14.88, *p*<.001, *η_p_^2^* = .05) (see Table 1). The Bonferroni-corrected analysis showed that tennis players (2703.79±349.85 Kcal/d) and swimmers (2761.±389.59 Kcal/d) had similar levels of physical activity, whereas both the athletic groups had higher levels of physical activity than the sedentary controls (2703.79±349.85 Kcal/d) [tennis players vs. sedentary controls, *t*(39) = 4.46, *p*<.001; swimmers vs. sedentary controls, *t*(39) = 4.95, *p*<.001].

#### Estimation of levels of aerobic fitness

There was a significant group difference for the estimated levels of aerobic fitness, (*F*(2,57) = 155.67, *p*<.001, *η_p_^2^* = .85) (see Table 1). The Bonferroni-corrected analysis showed that tennis players (55.60±2.40 ml/kg/min) and swimmers (55.05±2.41 ml/kg/min) had similar levels of aerobic fitness, whereas both the athletic groups had higher levels of aerobic fitness than the sedentary controls (43.85±2.32 ml/kg/min) [tennis players vs. sedentary controls, *t*(25) = 15.63, *p*<.001; swimmers vs. sedentary controls, *t*(25) = 14.90, *p*<.001].

### Filtered Responses

Here we didn’t find significant differences in wrong response rates for go trials, *F*(2, 57) = 2.17, *p* = .124, *η_p_^2^* = .07. [tennis players: 0.89±0.73%; swimmers: 0.82±0.87%; sedentary controls: 0.43±0.65%].

### Go RTs (No Stop-signal)

**Figure 4:**
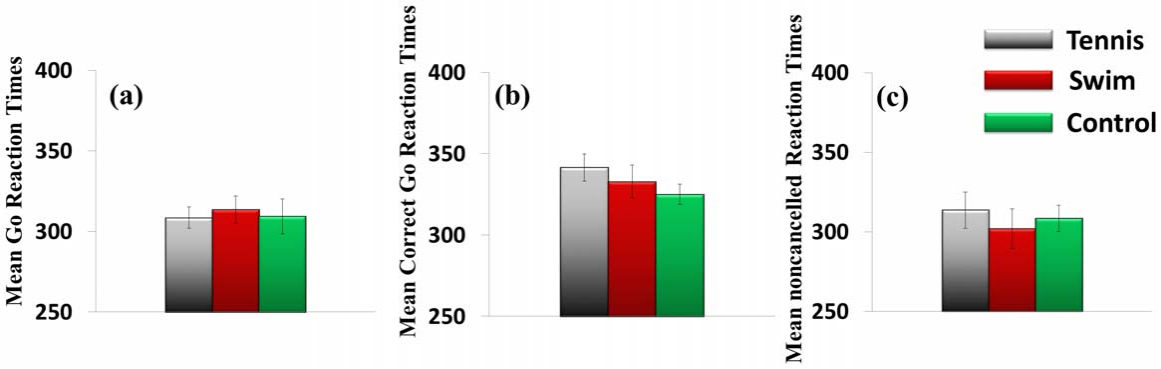
Mean Go RTs (in milliseconds) for each condition across tennis players, swimmers, and controls. (a) No stop-signal condition. (b) Correct Go RTs in stop-signal condition. (c) Noncancelled Go RTs in stop-signal condition. Each error bar shows the standard error of the mean.

Fig. 4 (a) shows the mean go RTs without stop signals. There were no significant differences across groups on this measure, *F*(2, 57) = 1.31, *p* = .278, *η_p_^2^* = .00 (tennis players: 308.55±25.67 ms, swimmers: 313.65±32.52 ms, sedentary controls: 309.35±41.76 ms).

### Go RTs (Correct Responses)

Fig. 4 (b) shows the correct mean go RTs in the stop-signal task. No group effects were observed, *F*(2, 57) = 1.31, *p* = .278, *η_p_^2^* = .04 (tennis players: 341.64±32.20 ms, swimmers: 332.80±39.14 ms, sedentary controls: 325.07±24.00 ms).

### Go RTs (Noncancelled Responses)

Fig. 4 (c) shows the mean go reaction times when responses were not inhibited appropriately. Again, there were no significant differences across groups, *F*(2, 57) = .41, *p* = .668, *η_p_^2^* = .01 (tennis players: 313.67±44.20 ms, swimmers: 302.04±48.34 ms, sedentary controls: 308.55±27.07 ms).

### Stop Signal Reaction Times

**Figure 5:**
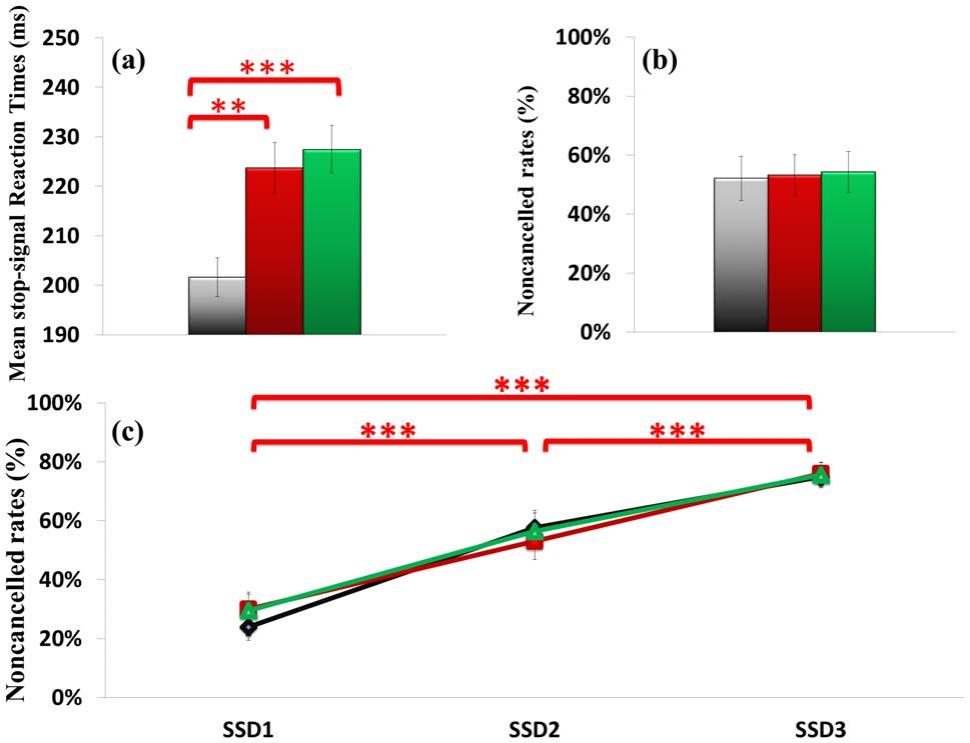
Inhibitory control performance across tennis players, swimmers and controls. (a) Mean stop-signal reaction times (b) Noncancelled rates (c) Inhibitory functions for each SSD. Each error bar shows the standard error of the mean. Note: **p<.01; ***p<.001.

Fig. 5 (a) shows the means SSRTs for the different groups. The main effect of the group factor was significant, *F*(2, 57) = 11.81, *p*<.001, *η_p_^2^* = .29. Results of *post-hoc* analysis showed that tennis players (201.64±15.16 ms) had significantly shorter SSRTs in comparison to swimmers [222.99±19.75 ms, *t*(39) = −3.76, *p* = .001] and to sedentary controls [227.47±18.65 ms, *t*(39) = −4.55, *p*<.001].

### Noncancelled Rates

Fig. 5 (b) shows the noncancelled response rates. No significant differences were observed among the noncancelled rates for the three groups, *F*(2, 57) = .08, *p* = .980, *η_p_^2^* = .00 (tennis players 0.52±0.14, swimmers 0.53±0.18, sedentary controls 0.54±0.14).

### Inhibition Function

Fig. 5 (c) shows the inhibition function across groups. The noncancelled rate was significantly increased with the increment of SSD, *F*(2, 114) = 279.31, *p*<.001, *η_p_^2^* = .83. However, no significant differences were found among groups, *F*(2, 57) = .02, *p* = .981, *η_p_^2^* = .00. The interaction effect between the SSD factor and the groups factor was not significant, *F*(2, 64) = 1.18, *p* = .325, *η_p_^2^* = .04.

### Hierarchical Stepwise Regression Analysis

Table 3 provides a summary of the hierarchical stepwise regression analysis. The overall regression model for the first step was significant, *R^2^* = .115, *F*(1, 59) = 7.53, *p* = .008, with only VO_2max_ included, suggesting the estimated VO_2max_ is most associated with SSRT compared to other basic factors (e.g., BMI, training experience, and estimated levels of physical activity). In addition, the second step was also significant, *R^2^* = .293, *F*(2, 59) = 11.81, *p*<.001, with a significant change to the model (△*R^2^* = .178, *F*(2, 57) = 14.35, *p*<.001), indicating TenCon was associated with greater modulation of SSRT relative to other factors (e.g., BMI, training experience, estimated levels of physical activity and VO_2_max).

**Table 3:** Results of hierarchical stepwise regression analysis (*n* = 60).

	Step 1	Step 2
	Basic factors	Group factors
VO_2max_	−.339**	−.100
BMI	−.010	.106
Training experiences	−.051	.070
Kilocalorie expenditure	−.053	−.041
TenCon	–	−.485***
SwimCon	–	−.055
R^2^	.115	.293
Adjusted R^2^	.100	.268
F	7.53**	11.81***
△*R^2^*	.115**	.178**
△*F*	7.53**	14.36***

Note: **p<.01;

*** *p*<.001; Entries represent standardized regression coefficients (β).

## Discussion

Previously, inhibitory control has been suggested to be superior in athletes when compared to non-athletes. The aim of the present study was to further determine whether this advantage is modulated differentially according to the nature of the sport undertaken as well as whether it can be seen in a non-sport specific cognitive task. Accordingly, we compared inhibitory control in athletes from an open skill sport (varsity tennis team), athletes from a closed skill sport (varsity swimming team) as well as sedentary controls. By using the stop signal task without a sport-specific design [1], [24], [28], an index of inhibitory control, as well as of impulsivity, was obtained from these groups.

The primary finding of the present study was that the stop signal reaction times (SSRTs) were significant shorter for the tennis players compared to swimmers and sedentary controls, whereas no difference were observed between the swimmers and sedentary controls. Subsequent hierarchical stepwise regression analysis showed that although the estimated VO_2max_ has the highest correlation with SSRT relative to other basic factors such as BMI, training experience, and estimated levels of physical activity in the first step, this effect became non-significant after the group factors (TenCon and SwimCon) were taken into account in the second step, with the results revealing that this effect is specifically a result of the TenCon factor. This suggests that the ability to inhibit prepotent responses benefited more from tennis training than from swimming training even though both groups shared similar estimated levels of aerobic fitness, perhaps due to different sets of cognitive or motor demands acquired for athletes in different categories of sports [17], [18], and this effect are less affected by other potential confounding factors. However, no effects on go RT with and without stop-signal was found, suggesting the effects of sport training is less pronounced on the execution of prepotent responses. In addition, the absence of a difference across groups in error rate may be due to the individual task difficulty, with our stop-signal task [24], [41] controlled to be consistent for each participant.

It is worthy of note, however, that there is a possibility that individuals with a better ability to inhibit prepotent responses may be more likely to pursue or be successful in sports where this is presumably of benefit, such as tennis. While we consider this to be unlikely and the difference seen to be a consequence of training, it would nevertheless be beneficial for future study to systematically test this. However, previously it has been shown that exercise-training affects inhibitory control [35]. This study demonstrated that children with DCD developed more efficiency in inhibitory control after a 10-week table tennis training program. Training-induced enhancement in other cognitive functions have also be seen by training in different domains, for example, following video game training [42]. Li et al. [42] found that contrast sensitivity in young adults was improved via a 50-hour action video game training regimen.

Prior experiments employing Go/No-go tasks or anti-saccade tasks have shown superior inhibitory control in athletes [7]–[9], [11], [13]–[16]. In these studies, athletes from open skills sports such as baseball players, basketball players, table tennis players, or fencers were demonstrated to react faster and commit fewer errors than non-athletic controls during cognitive tasks requiring response inhibition. Similar to this, the present study using a stop-signal task observed greater inhibitory control in tennis players when compared to the sedentary controls, as shown by shorter SSRTs, indicating a shorter time was required for tennis players to withhold their prepotent actions.

In contrast with previous findings, we observed no inhibitory control advantage in swimmers, who showed similar SSRTs to sedentary controls, despite the estimated levels of physical activity and aerobic fitness being significant different between the two groups. Previously shortened SSRTs have been reported in elderly non-athletes following aerobic training [5], resulting in the claim that aerobic exercise enhances higher-order cognitive functions, including inhibitory control. There are some possible reasons for this discrepancy. Firstly, age-related changes in cognitive functioning might interact with any beneficial effects of training to different degrees. It may be that there is little room for exercise-related facilitation during cognitive health peaks [6], meaning that they are much less likely to be observed in young subjects. Similar to this argument, Scisco et al. [43] found no difference in executive control between young adults with different levels of aerobic fitness. Secondly, inhibitory control might benefit more from a combination of aerobic and skill training than aerobic training alone in young subjects. Chan et al. [7] found that there was no performance difference on a Go/No-go task between average-fitness fencers and average-fitness non-athletes. However, high-fitness fencers showed significant fewer commission errors compared to high-fitness non-athletes. In line with this, Di Russo et al. [20] reported that individuals with physically disability benefit more in executive control from open skill sports (wheelchair baseball) relative to close skill sports (swimming).

Moreover, our results show a difference in inhibitory control between athletes with similar levels of estimated physical activity and VO2max from different sport categories, further indicating the importance of comparing different athlete type [4], [18], [22]. Importantly, the current study provides additional evidence for this argument in inhibitory control, which still required further examination [4]. Thus, the data suggests that sports involved highly cognitive demands may develop superior inhibitory control with respect to sports in which the training environment is highly consistent, predictable and self-paced for players. In addition, the similar performance of swimmers and sedentary controls relative to tennis players doesn’t mean aerobic fitness is of less importance for enhancement in inhibitory control. For example, Chan et al. [7] found high-fitness fencers showed superiority in inhibitory control relative to non-athletes whereas low fitness fencers did not, indicating the importance of combination of physical and cognitive skill components for cognitive improvement. Other dependent variables, such as go RTs, which represent a measure of impulsivity, were comparable across groups. This might be due to responses which do not require executive control being less facilitated by engagement in sport training [5], [6]. In agreement with this, other studies have also shown no difference in simple responses between athletes and non-athletes [8], [9], [11], but see [10]. This pattern of results helps to rule out the possibility of shorter SSRT for tennis players being a simple consequence of a group difference processing speed. Similar findings have been found in a study comparing patients with Parkinson’s disease and healthy controls using a stop-signal task. It was found that both the groups had comparable go RTs but prolonged SSRTs were found in patients [32]. These findings are also consistent with the pattern seen with transcranial magnetic stimulation (TMS) in which elevated SSRTs were seen but go RTs were unaffected by TMS delivered over right frontal eye field [23]. Therefore, the present data suggests that the facilitative mechanism on inhibitory control resulting from sport training may be independent of the processing of impulsive responses. Further studies are required to shed light on this issue.

Our findings may challenge previous studies exhibiting an athletic advantage in inhibitory control only when the task used was sport-specific (for instance, strike-zone-like stimulus-response design for baseball players [8], [9]). Kida et al. [9] found that baseball players, but not tennis players, responded faster than non-athletes in a Go/No-go task with a baseball-specific design. Additionally, Nakamoto and Mori [8] showed baseball players had faster Go/No-go RTs compared to their athletic control selectively in the baseball batting-specific condition but not in other non-baseball-specific conditions. In contrast with these results, the present study showed an athletic advantage in inhibitory control by using a standard stop-signal task with no sport-specific design selectively for open skill sports [23], [24], suggesting sport-related enhancements can still be seen outside of a sport-specific context depending on the sport type. This corresponds with the previous literature using meta-analysis finding an athletic advantage in laboratory cognitive tests without employing sport-specific designs, especially for open skill sports [4]. Interestingly, our findings are also in line with the claim that there is a connection between sport and the ability to perform mental image transformations, and this ability may not necessary be sport specific [44]. This may also support the concept of motor cognition, which views the motor system as participating in mental processing [45]. Indeed, athletes from open skill sports (e.g., racket sports) are required to process information in a rapidly changing and unpredictable environment, which might lead to superior performance of interceptive actions, hand-eye coordination and perception-action [33] or improve inhibition of inappropriate movements or response selection, and this may also resulting in developing more flexible visual attention, decision making and action execution [21] with respect to closed skill sports such as swimming. Accordingly, the present study suggests that this enhancement from open sport training may transcend the sport-specific context at young ages.

While the present findings shed light on the group differences between athletes of different sports categories and non-athletes on inhibitory control, there are some limitations to the interpretations that require caution. For example, the levels of physical activity and aerobic fitness in the present study are based on estimation not measurement. The methodological differences might bias the present findings compared to other studies adopting direct measurement of VO2max [5]. Therefore, more direct evaluation of aerobic fitness or physical activity is warranted for future studies. In addition, although we controlled the physiological characteristics, such as height, weight and BMI which may bias the effect of sport on inhibitory control [36], to be similar across groups, other indices such as % body fat or % muscle may be appropriate factors being taken into account in the comparison between athletes and non-athletes. Moreover, we only recruited male participants, thus, the results might differ in females or in a mixed sample [4]. Despite the fact that men and women show no differences at a behavioral level during a stop-signal task [46], it may be of interest to test the interaction between sporting training and gender effects on inhibitory control.

In conclusion, to the best of our knowledge, the present study is the first to demonstrate the difference in inhibitory control in young athletes from different sport categories as well as non-athletes by utilizing the stop-signal task without a sport-specific design. Our data showed inhibitory control is superior for tennis players compared to swimmers and non-athletes, suggesting fundamental cognitive control might benefit more from training in open skill sports. On the other hand, measures of simple impulsive responses were not significantly different across groups. These finding may have important practical and theoretical implications. First, sport with both physical and cognitive demands may provide a potential clinical intervention for those who have difficulties in inhibitory control, such as patients with ADHD [31], Parkinson’s disease [32], or DCD [35]. Especially for patients with Parkinson’s disease, whose later pathological stage involves motoric effects, sports may be a fruitful approach for early intervention. Second, as we found that groups differed selectively in the index of inhibitory control rather than in impulsivity, further studies should try to explore whether the beneficial effects on cognitive function from sport interact differently with these two processes.
